# Hypoglycemia in type 1 diabetes: a burden to worry about during treatment

**DOI:** 10.20945/2359-3997000000574

**Published:** 2022-11-28

**Authors:** Sergio Atala Dib

**Affiliations:** 1 Universidade Federal de São Paulo Escola Paulista de Medicina Departamento de Medicina São Paulo SP Brasil Centro de Diabetes e Endocrinologia, Divisão de Endocrinologia e Metabolismo, Departamento de Medicina, Escola Paulista de Medicina, Universidade Federal de São Paulo, São Paulo, SP, Brasil

Since the discovery of insulin in 1921 and with the evolution of different insulin analogs, infusion subcutaneous systems and glycemic monitoring devices, patients with type 1 diabetes have been experimenting a significant improve in quality of life and living longer with fewer complications.

Nevertheless, a next drawback to be solved in the current insulin therapy, which is usually made through multiple daily injections (MDI) or by continuous subcutaneous insulin infusion (CSII) using an insulin external pump, is to mimic endogenously secreted insulin by subcutaneous insulin. Through the last approach mentioned here, we invariably submitted the patient to peripheral hyperinsulinemia, weight gain, and risk of hypoglycemia.

Hypoglycemia is defined by the American Diabetes Association (ADA) Workgroup on Hypoglycemia (1) as “all episodes of abnormally low plasma glucose concentration that expose the individual to potential harm”. This definition is ample and involves episodes of asymptomatic hypoglycemia where the defenses against subsequent hypoglycemia are impaired (2). At biochemical level, this group proposes that any blood glucose value equal to or below 70 mg/dL (3.9 mmol/l) should represent hypoglycemia, with or without symptoms, as this is the glycemic level at which hormonal counter-regulation is activated in non-diabetic adults (3) and that frequent periods of this glycemic levels can reduce the secretion of the counter regulatory hormones as glucagon and adrenaline in response to a next episode of hypoglycemia (4).

In general, at clinical type 1 diabetes phase, the first and second physiological defenses against hypoglycemia decrease in insulin and increase in glucagon, and the third physiological defense, an increase in adrenaline, is often decreased as well (5). The loss of glucagon response (6) is related to the degree of beta-cell failure found at type 1 diabetes. As you know, the residual endogenous insulin secretion (C-peptide-positivity) is a protective factor (7), although it can be also triggered by a recent history of hypoglycemia (8), prior exercise or sleep (9). On the other hand, the mechanism of decrease on the sympathoadrenal response to hypoglycemia in type 1 diabetes is not well known, but may involve the brain and afferent or efferent components of the sympathoadrenal system. These alterations are associated with a 25-fold (10) increase of severe iatrogenic hypoglycemia. Exposure to several episodes of hypoglycemia can accentuate this defect and it may develop a change in the symptom's complex that characterize hypoglycemia, resulting in a clinical phenomenon referred to as impaired awareness of hypoglycemia (IAH). We have data (11) confirming the relationship between unawareness of hypoglycemia and the number of severe hypoglycemia episodes observed in individuals with type 1 diabetes. In these data we also shown that older age, duration of diabetes and low estimated glomerular filtration rate (eGFR) were associated with unawareness of hypoglycemia. IAH is a gradually developing phenomenon and it's also known as hypoglycemia-associated autonomic failure (HAAF) (12). It is clinical important to be aware that in a significant percentage of patient, 2-3 weeks of rigorous avoidance of hypoglycemia can reverse hypoglycemia unawareness and improve the deficient epinephrine component of defective glucose counter regulation (13).

Observational studies have reported that non-severe hypoglycemia frequency ranges between 3.5-7.2 events/month in type 1 diabetes patients (14). Meanwhile, severe hypoglycemia can occur in 30% to 40% of these subjects annually in a mean of 1 to 1.7 episodes per patient per year (15).

The clinical implications of hypoglycemia can be divided in short-term and long-term effects. Short-term effects include: cognitive impairment, mood change, impairment of work and driving performance, impairment of social, sport and travel activities, hypothermia, falls, cardiovascular and cerebrovascular events, seizures, and coma. The long-term implications are: fear of hypoglycemia, reduced quality of life, weight gain, possible worsening of diabetic chronic complications, acquired hypoglycemia-induced syndromes, cognitive decline and possible onset or acceleration of dementia (16). As hemodynamic responses to hypoglycemia we have heart beat increase, increased systolic blood pressure, decreased diastolic blood pressure, increased cardiac output, increased myocardial contractility, electrocardiographic changes as T-wave flattening or inversion, S-T- interval depression and QT-interval prolongation (17). Hypoglycemia can be fatal and data shown that more than 8% of the deaths can be directly related to hypoglycemia in type 1 diabetes subjects younger than 56 years (18).

Most of the epidemiology of hypoglycemia in type 1 diabetes patients come from westernized societies and it might not be relevant to population in other parts of the world where ethnic, cultural and healthcare organizational aspects are different. Therefore, the publication of the results of a cross-sectional, multicenter study showing the epidemiology and the risk factors of hypoglycemia in subjects with type 1 diabetes in Brazil, in this issue of *Archives of Endocrinology and Metabolism,* by de Souza and cols. (19), will collaborate for the analyses of the reasons for the differences in hypoglycemia rates among the global regions, offering us data to reduce this complex clinical problem during insulin therapy in type 1 diabetes patients.

This study was conducted between August 2011 and August 2014 (for 3 years) in 14 public secondary and tertiary health care clinics in 10 Brazilian cities, from all five main geographic regions of the country. A large sample (n=1760) of individuals (aged > 13 y.o.) with type 1 diabetes were followed, at each center, for at least 6 months. They were under the care of an endocrinologist from the Brazilian Unified Health Care System (SUS). Other clinical variables, such as the presence of chronic diabetes complications, frequency of self-monitoring of blood glucose (SMBG), insulin therapeutic regimen (ITR), self-reported frequency of hypoglycemia in the previous month, besides any hospitalization, were recorded. Hypoglycemia was considered when blood glucose levels was ≤ 70 mg/dL (3.9 mmol/l) or required third-party help to recover from hypoglycemia during the last month. Furthermore, these patients were divided on no severe hypoglycemia (NSH), when they recovered of the event by themselves, and severe hypoglycemia (SH), when they needed assistance for clinical recovery. Individuals who did not report any hypoglycemic episode in the previous month were included in the non-hypoglycemic group (NHG). The mean age of the type 1 diabetes group was 30.0 ± 11.9 years, duration of diabetes of 14.5 ± 9.4 years and the mean HbA1c was 9.0 ± 2.1%. The hypoglycemic group (HG) had longer duration of diabetes, lower HbA1c, used more antidepressants drugs, the monthly average family income was higher, had more access to supplementary health care, and longer formal education than NHG group. On respect to insulin therapy, the HG received lower total daily insulin supplementation(U/kg/day), less prandial insulin, equal basal insulin, more rapid-acting prandial insulin and long-acting basal insulin analogs, more self-monitoring blood glucose as they were using more continuous subcutaneous insulin infusion than the NHG group. Interestingly, the HG group had more ketoacidosis at the diabetes onset than the NHG group. In relation to diabetes chronic complications, the HG group had more retinopathy, lower estimated glomerular filtration rate (eGFR ml/min) and similar sensorimotor neuropathy, as cardiovascular autonomic neuropathy (CAN), than the NHG group. During the previous month, the patients from the intra hypoglycemic group had at least one episode of severe hypoglycemia (19%), nocturnal hypoglycemia (24.3%) or asymptomatic hypoglycemia (38.8%). The last patients (asymptomatic hypoglycemic subgroup) showed more prevalence (39.1%) of cardiovascular autonomic neuropathy (CAN) than the group with SH (33.2%, p = 0.03). The main features related to SH were higher HbA1c, higher alcohol consumption, lower monthly average family income and access to supplementary health care, less use of long-acting basal insulin analog, higher hospitalization in the previous year and higher prevalence of microvascular complication, such as retinopathy, diabetic kidney disease, sensorimotor neuropathy, and CAN, compared to the group with no severe hypoglycemia. It is important to note that although CAN wasn't more prevalent when they compared the non-hypoglycemic group to hypoglycemic group as a total, CAN is more prevalent on the group with severe (42.9%) than in the group with no severe (33.7%, p<0.01) hypoglycemia.

The present Brazilian study has shown a prevalence of hypoglycemia of 74.9%. This result is like the one found in a global HAT study (20) involving 24 countries, published some years ago, where the primary endpoint was the percentage of patients experiencing at least one hypoglycemic event during the 4-week follow-up period (having a prevalence of 73.4% in 7108 type 1 diabetes patients). The group had a similar duration of the disease (12 years), but the subjects were was younger (12.0 y.o.) and had a better HbA1c (7.9% vs 9.0 + 2.1%) than in the Brazilian study. In this global (HAT) study, the subgroup from Latin America (n = 427; Argentina and Mexico) have shown a higher prevalence of hypoglycemia (93.9% vs 74.9%) and less prevalence of nocturnal hypoglycemia (17.7% vs 24.3%) than the Brazilian study. It is important to note that in this global (HAT) study the hypoglycemia rates were only weakly associated with HbA1c level in the type 1 diabetes group, as 83.8% with HbA1c < 7.0%, 85% with HbA1c, 7%-9% and 79.6% with HbA1c > 9.0%, respectively. These can be explained by the fact that the HbA1c cannot detect the glycemic fluctuations ant the magnitude, and frequency of daily fluctuations of blood glucose levels can be correlated to frequency of hypoglycemia episodes (21).

Other important aspect to remember in the studies about epidemiology of hypoglycemia episodes is that data about severe hypoglycemia for up to 1 year after an event has occurred is more robust than mild hypoglycemics episodes, since identification of asymptomatic events requires frequent blood glucose monitoring. These can observed in this Brazilian study, where self-monitoring of blood glucose is more prevalent in the hypoglycemic group (96.4%) than in the non-hypoglycemic group (88.9%), but it was similar in the groups with no severe hypoglycemia and at least one episode of severe hypoglycemia. The use of continuous glucose monitoring (CGM), which measures glucose levels in the interstitial space, although discrepancies can exist between interstitial and capillary blood glucose measurements, particularly during the night, has demonstrated that asymptomatic and unrecognized hypoglycemia (22) is frequent in different forms of insulin therapy.

Basal insulin is a key component of insulin therapy in type 1 diabetes and the present Brazilian study (19) was able to show the value of this part of insulin therapy that, at higher doses, was associated to more prevalence of severe hypoglycemic episodes.

Finally, weekly alcohol consumption in the Brazilian study (19) demonstrated an odds ratio of 5.64 to severe hypoglycemia, after final adjusted logistic regression analysis with SH, as the dependent variable. In the existing literature, the rates of alcohol consumption by type 1 diabetes younger adults range from 19.3% to 26% (23). Eighty percent of the ingested alcohol is metabolized in the liver, leading to an increase in the so-called “redox shift” (increase in the NADH: NAD ratio) resulting in the inhibition of gluconeogenesis (24). Gluconeogenesis is required to maintain glucose levels in the fasting state and inhibition of gluconeogenesis may result in hypoglycemia. Glycogenolysis is also impaired by alcohol (25) and it can also collaborate to hypoglycemia when associated to situations where the individuals have glycogen stores depleted, as malnourished, alcoholics, very low carbohydrate diets, fasting or drinking without consuming food (26).

In summary, this Brazilian study corroborated to other similar trials, demonstrating that hypoglycemia continues to occur in type 1 diabetes individuals despite the implementation of advanced diabetes technologies. The knowledge of the main factors around hypoglycemia, besides the innovative research to provide plasma glucose regulated insulin replacement and secretion, will decrease the barriers of hypoglycemia during insulin treatment of type 1 diabetes and improve the quality of life.

## References

[1] Workgroup on Hypoglycemia, American Diabetes Association (2005). Defining and reporting hypoglycemia in diabetes: a report from the American Diabetes Association Workgroup on Hypoglycemia. Diabetes Care.

[2] Cryer P (2008). The barrier of hypoglycemia in diabetes. Diabetes.

[3] Mitrakou A, Ryan C, Veneman T, Mokan M, Jenssen T, Kiss I (1991). Hierarchy of glycemic thresholds for counterregulatory hormone secretion, symptoms, and cerebral dysfunction. Am J Physiol.

[4] Davis SN, Shavers C, Mosqueda-Garcia R, Costa F (1997). Effects of differing antecedent hypoglycemia on subsequent counterregulation in normal humans. Diabetes.

[5] Cryer PE (2006). Mechanisms of sympathoadrenal failure and hypoglycemia in diabetes. J Clin Invest.

[6] Gerich JE, Langlois M, Noacco C, Karam JH, Forsham PH (1973). Lack of glucagon response to hypoglycemia in diabetes: evidence for an intrinsic pancreatic alpha cell defect. Science.

[7] Mühlhauser I, Overmann H, Bender R, Bott U, Berger M (1998). Risk factors of severe hypoglycaemia in adult patients with Type I diabetes--a prospective population based study. Diabetologia.

[8] Heller SR, Cryer PE (1991). Reduced neuroendocrine and symptomatic responses to subsequent hypoglycemia after 1 episode of hypoglycemia in nondiabetic humans. Diabetes.

[9] Cryer PE (2008). The Barrier of Hypoglycemia in Diabetes. Diabetes.

[10] White NH, Skor DA, Cryer PE, Levandoski LA, Bier DM, Santiago JV (1983). Identification of type I diabetic patients at increased risk for hypoglycemia during intensive therapy. N Engl J Med.

[11] Paes T, Rolim LC, de Sa JR, Dib SA (2020). Awareness of hypoglycemia and spectral analysis of heart rate variability in type 1 diabetes. J Diabetes Complications.

[12] Dagogo-Jack S, Rattarasarn C, Cryer PE (1994). Reversal of hypoglycemia unawareness, but not defective glucose counterregulation, in IDDM. Diabetes.

[13] Cranston I, Lomas J, Maran A, Macdonald I, Amiel SA (1994). Restoration of hypoglycaemia awareness in patients with long-duration insulin-dependent diabetes. Lancet.

[14] Östenson CG, Geelhoed-Duijvestijn P, Lahtela J, Weitgasser R, Markert Jensen M, Pedersen-Bjergaard U (2014). Self-reported non-severe hypoglycaemic events in Europe. Diabet Med.

[15] McCrimmon RJ, Sherwin RS (2010). Hypoglycemia in type 1 diabetes. Diabetes.

[16] Frier BM (2014). Hypoglycaemia in diabetes mellitus: epidemiology and clinical implications. Nat Rev Endocrinol.

[17] Wright RJ, Frier BM (2008). Vascular disease and diabetes: is hypoglycaemia an aggravating factor?. Diabetes Metab Res Rev.

[18] Gagnum V, Stene LC, Jenssen TG, Berteussen LM, Sandvik L, Joner G (2017). Causes of death in childhood-onset Type 1 diabetes: long-term follow-up. Diabet Med.

[19] de Souza ABC, Correa-Giannella MLC, Gomes MB, Negrato CA, Nery M (2022). Epidemiology and risk factors of hypoglycemia in subjects with type 1 diabetes in Brazil: a cross-sectional, multicenter study. Arch Endocrinol Metab.

[20] Khunti K, Alsifri S, Aronson R, Cigrovski Berković M, Enters-Weijnen C, Forsén T (2016). HAT Investigator Group. Rates and predictors of hypoglycaemia in 27 585 people from 24 countries with insulin-treated type 1 and type 2 diabetes: the global HAT study. Diabetes Obes Metab.

[21] Monnier L, Wojtusciszyn A, Colette C, Owens D (2011). The contribution of glucose variability to asymptomatic hypoglycemia in persons with type 2 diabetes. Diabetes Technol Ther.

[22] McGowan K, Thomas W, Moran A (2002). Spurious reporting of nocturnal hypoglycemia by CGMS in patients with tightly controlled type 1 diabetes. Diabetes Care.

[23] Barnard K, Sinclair JM, Lawton J, Young AJ, Holt RI (2012). Alcohol-associated risks for young adults with Type 1 diabetes: a narrative review. Diabet Med.

[24] Krebs HA, Freedland RA, Hems R, Stubbs M (1969). Inhibition of hepatic gluconeogenesis by ethanol. Biochem J.

[25] van de Wiel A (2004). Diabetes mellitus and alcohol. Diabetes Metab Res Rev.

[26] White ND (2017). Alcohol Use in Young Adults With Type 1 Diabetes Mellitus. Am J Lifestyle Med.

